# Unraveling causal links between chronic rhinosinusitis and peripheral artery diseases: insights from genetic correlations through genome-wide association studies

**DOI:** 10.1016/j.bjorl.2026.101891

**Published:** 2026-09-02

**Authors:** Bo Wei, Jiaoyu He, Weigang Gan, Binbin Wang, Feng Liu

**Affiliations:** a Sichuan University, West China Hospital, ENT Department, Chengdu, China; b Chengdu Second People’s Hospital, Renji Medical Research Center, Chengdu, China

**Keywords:** Chronic rhinosinusitis, Cardiovascular diseases, Peripheral artery diseases, Genetic correlation, Causal relationship

## Abstract

**Objectives:**

Chronic Rhinosinusitis (CRS) shares epidemiological links with Cardiovascular Diseases (CVDs), however, their shared genetic basis remains unclear. We hypothesized that pleiotropic genetic variants underlie CRS-CVDs links via distinct biological pathways.

**Methods:**

Using large-scale GWAS data from European-ancestry individuals, we assessed global and local genetic correlations. We applied Genomic Structural Equation Modeling (Genomic SEM) to dissect shared genetic architecture, performed bidirectional Mendelian Randomization (MR) to infer causality, and conducted cis-eQTL colocalization to identify shared genetic signals. Finally, in vitro endothelial models (HUVECs) validated the functional dynamics of candidate genes under CRS-mimicking inflammatory stress.

**Results:**

CRS showed significant genetic correlations with multiple CVDs. Genomic SEM revealed a latent factor structuring shared genetic risk through three pathways: artery diseases, myocardial diseases, and heart failure. Local genetic correlations identified significant local genetic correlations specifically between CRS and Peripheral Atherosclerosis (PAS)/Peripheral Artery Disease (PAD) specifically within the chr6: 31.57‒33.24 Mb locus. MR demonstrated causal effects of CRS on PAD (OR = 1.23, p = 0.022) and PAS (OR = 1.21, p = 0.011), but not vice versa. Genetically predicted HLA-DRB1, APOM, and COL11A2 expression conferred protection, while HLA-DQA2 increased risk. Crucially, in vitro validation corroborated these pathogenic trajectories, inflammatory stress significantly downregulated the protective APOM and upregulated the risk-associated HLA-DQA2 alongside pro-atherogenic VCAM-1, while HLA-DRB1 exhibited a compensatory upregulation (p < 0.05).

**Conclusion:**

CRS shares global genetic liability with CVDs, structured through three primary etiological pathways. Causal effects of CRS on peripheral artery diseases are mediated by immune and lipid-related genes within the chr6 locus, revealing divergent pleiotropic mechanisms. Our integrated genetic and in vitro evidence provides a mechanistic framework wherein chronic mucosal inflammation contributes to systemic endothelial vulnerability, thereby highlighting candidate targets for mechanism-directed therapy.

## Highlights

• Genetic correlations link Chronic Rhinosinusitis (CRS) with multiple Cardiovascular Diseases (CVDs).
• A chromosome 6 locus shows local genetic overlap between CRS and Peripheral Artery Diseases (PADs).
• Mendelian Randomization (MR) reveals causal effects of CRS on PADs risk.
• HLA-DRB1 and APOM gene expression confers protection against both CRS and PADs.
• Network analysis implicates immune, metabolic, and inflammatory pathways in CRS-CVDs links.

## Introduction

Chronic Rhinosinusitis (CRS) is a prevalent inflammatory disorder of the nasal and paranasal sinuses, affecting approximately 10% of the global population and imposing substantial healthcare burdens through persistent symptoms, reduced quality of life, and recurrent medical utilization.^1^, ^2^, ^3^ Although traditionally regarded as a localized condition, emerging epidemiological studies suggest systemic implications, particularly an increased risk of Cardiovascular Diseases (CVDs).^4^ Observational evidence indicates that CRS patients exhibit higher rates of hypertension, atherosclerosis, and myocardial infarction compared with non-CRS cohorts.^5^, ^6^, ^7^ However, the causal mechanisms underlying this association remain poorly understood, with debates centering on whether shared genetic factors, systemic inflammation, or secondary effects of CRS treatment drive this comorbidity.

CVDs, encompassing Ischemic Heart Disease (IHD), Atherosclerosis (AS), Heart Failure (HF), Myocardial Infarction (MI), and Peripheral Artery Disease (PAD), account for more than 17-million deaths annually and represent the leading cause of mortality worldwide.^8^ Established risk factors such as hypertension, dyslipidemia, and diabetes do not fully explain for the excess cardiovascular risk observed in CRS patients.^9^^,^^10^ Chronic inflammation ‒ a hallmark of CRS ‒ promotes endothelial dysfunction, oxidative stress, and atherosclerosis, providing plausible biological pathways linking sinonasal inflammation to cardiovascular pathology.^11^, ^12^, ^13^ Nevertheless, confounding factors in observational studies, including smoking, obesity, and corticosteroid use, complicate causal inference.^14^ Thus, disentangling genetic and causal relationships is critical for understanding pathophysiological links and identifying targeted interventions.

LD Score Regression (LDSC) enables quantification of genome-wide genetic correlations, offering an initial screen for shared polygenic effects across traits.^15^ Genomic Structural Equation Modeling (Genomic SEM) dissects shared genetic architectures across traits by modeling latent factors, uncovering etiological pathways not evident in univariate analyses.^16^ To resolve these broad correlations into localized effects, local genetic correlation analysis (ρ-HESS) identifies region-specific pleiotropic loci, revealing genomic hotspots where shared genetic architecture drives comorbidity.^17^ Additionally, Mendelian Randomization (MR) leverages genetic variants as instrumental variables to infer causal relationships from observational data, minimizing confounding and reverse causation biases inherent in traditional epidemiological studies.^18^ This approach, analogous to randomized controlled trials in its use of genetic “randomization”, has successfully elucidated causal roles of biomarkers in cardiovascular pathogenesis.^19^ Finally, expression Quantitative Trait Loci (cis-eQTL) – based MR bridges genetic associations to functional consequences by testing whether cis-regulated gene expression mediates disease risk.

Taken together, this study applied an integrative genetic framework to elucidate the mechanistic links between CRS and CVDs. First, we quantified global genetic correlations using LD score regression and modeled shared genetic liability via hierarchical factor analysis with Genomic SEM. Second, we identified region-specific genetic overlaps through ρ-HESS. Subsequently, bidirectional MR reinforced by rigorous sensitivity analyses was performed to infer definitive causal relationships, followed by cis-eQTL colocalization to pinpoint specific upstream regulatory genes. Finally, to bridge computational inference with biological reality, we functionally validated our key genetic findings using an in vitro model. By combining statistical genetics with functional pathology, this approach systematically clarifies whether CRS-CVD comorbidity arises from shared architecture or unidirectional causality, ultimately providing mechanism-based actionable targets for precision prevention strategies.

## Methods

### Data sources

Summary-level GWAS data for CRS and CVDs were sourced from the UK Biobank and FinnGen consortium (n = 2,100,534; Supplemental Tables 1, 2). The publicly available data, accessed on 1 December 2024, comprise aggregated statistics with no individual-level identifiers. Details on genotyping, imputation, and quality control are described in original publications. Blood cis-eQTL data for 2,525 druggable genes were obtained from the eQTLGen Consortium (n = 31,684, European ancestry).^20^ Variants within ±1 Mb of each gene and with a Minor Allele Frequency (MAF) > 0.01 were analyzed.

### Linkage disequilibrium score regression

We assessed the heritability and genetic correlation between CRS and CVD subtypes using bivariate Linkage Disequilibrium Score Regression (LDSC). Analysis was restricted to European-ancestry summary statistics from the 1000 Genomes Project to ensure consistent LD reference. The baseline-LD model was applied to account for confounding factors.^21^

### Genomic structural equation modeling

Genomic SEM was employed to model the shared genetic architecture.^22^ LDSC first estimated the genetic covariance matrix between CRS and various CVD traits, thereby quantifying genetic overlap. Then, Exploratory and Confirmatory Factor Analysis (EFA, CFA) were performed to identify latent factors representing shared genetic liability. Model fit was deemed acceptable with a Comparative Fit Index (CFI) > 0.90.^16^

### Identifcation of local genetic correlations

Local genetic correlations were estimated using the ρ-HESS method, which computes SNP-based heritability and genetic covariance within approximately independent LD blocks (defined by Berisa and Pickrell).^23^^,^^24^ Significance was assessed using a block-jackknife procedure, with multiple testing correction via Bonferroni method.

### Bidirectional mendelian randomization analysis

Bidirectional two-sample MR was performed to test for causal relationships. Genetic instruments for the exposure were selected as SNPs associated at p < 5 × 10^−8^ (clumped with *r*^2^ < 0.001, distance < 10,000 kb), excluding rare (effect allele frequency < 10%) and ambiguous variants. The Inverse-Variance Weighted (IVW) method was primary, supplemented by weighted median and MR-Egger regression.^25^^,^^26^ Sensitivity analyses included tests for pleiotropy (MR-Egger intercept) and heterogeneity (Cochran’s *Q*).^27^ Additionally, the Mendelian Randomization Pleiotropy RESidual Sum and Outlier (MR-PRESSO) test was performed to detect and correct for horizontal pleiotropy and identify potential outlier SNPs.^28^ Reverse MR analysis was conducted similarly.

### Bayesian colocalization analysis

To assess evidence for shared causal variants, Bayesian colocalization was performed using the coloc R package (v5.2.1) for regions ±500 kb around significant SNPs.^29^ Analyses computed posterior probabilities for five hypotheses (H0‒H4). A combined posterior probability (PP.H3 + PP.H4) > 0.8 was considered evidence of colocalization.

### Construction of protein-protein interaction networks

To explore the functional landscape of the candidate genes, Protein-Protein Interaction (PPI) network was constructed using the STRING database (https://www.string-db.org/),^30^ with a confidence interaction score threshold set at 0.4 to establish significance. The construction and analysis of the PPI network were conducted using Cytoscape (v3.10.3) software.^31^

### In vitro inflammatory stimulation model

#### Cell culture

Human Umbilical Vein Endothelial Cells (HUVECs) (Pooled, Lonza) were cultured in complete growth medium consisting of Dulbecco's Modified Eagle Medium (DMEM, Gibco) supplemented with 10% fetal bovine serum (FBS, Gibco) and 1% Penicillin-Streptomycin solution (Gibco). Cells were maintained at 37 °C in a humidified atmosphere of 5% CO_2_. For subculturing, cells at 80%‒90% confluence were detached using 0.25% Trypsin-EDTA (Gibco) and passaged at a 1:3 ratio. Cells in the logarithmic growth phase were harvested and seeded into 6-well plates at an initial density of 30%‒40% (2 mL per well) for subsequent experiments.

#### Cytokine stimulation

Following a 12-h incubation period to allow for complete cellular adherence, the culture medium was replaced. The cells were randomly divided into two groups: 1) The Control group, which was maintained in standard complete DMEM, and 2) The Inflammatory Stimulation group. Based on established protocols for inducing endothelial dysfunction and inflammatory cascades,^32^^,^^33^ cells in the stimulation group were treated with 10 ng/mL of recombinant human Tumor Necrosis Factor-alpha (TNF-α) and 10 ng/mL of Interferon-gamma (IFN-γ) dissolved in complete medium. Both groups were incubated for 48 h prior to downstream analysis.

### RNA extraction and quantitative real-time PCR (qPCR)

Total RNA was isolated from 10^6^ human cells using the Animal Total RNA Isolation Kit (Foregene, China) following the manufacturer’s protocol. The concentration and purity of the RNA were measured using a K2800 nucleic acid analyzer. First-strand cDNA was synthesized from 1 μg of total RNA using the SweScript RT I First Strand cDNA Synthesis Kit (Servicebio, China) in a 20 μL reaction volume.

Quantitative PCR was conducted on a CFX Connect Real-Time System (Bio-Rad, USA) using SYBR Green qPCR Master Mix (Servicebio, China). Gene-specific primers were synthesized by Sangon Biotech (Shanghai, China). The sequences are listed in Table 1. The thermal cycling parameters were initial denaturation at 95 °C for 5 min, followed by 40 cycles of 95 °C for 10 s and 60 °C for 30 s. A melting curve analysis was conducted from 60 °C to 95 °C. All reactions were performed in triplicate. Relative mRNA expression was quantified using the 2^-ΔΔCt^ method, with the target gene levels normalized to the internal control.

**Table 1:** Sequences of primers used in RT-qPCR.

Gene	Forward Primer (5'‒3')	Reverse Primer (5'‒3')
β-Actin	AATCTGGCACCACACCTTCTACAA	GGATAGCACAGCCTGGATAGCAA
APOM	CCACTTCCGTTTCCCCGATA	CTCTGGGAATGTTTGGGGCT
VCAM-1	GGACCACATCTACGCTGACA	TTGACTGTGATCGGCTTCCC
HLA-DRB1	CAGGACCTGGTTGCTACTGG	GGCAAAGGGGAGCACAAAAG
HLA-DQA2	TCAGGGCTGACTGAGAGCATA	CAAGGGCAGAGGGTTCGTTG
COL11A2	GATCTCCACAGCAGCAACCAT	TGGGTGTCTTCCCTCTCTGGG

## Results

### Global genetic correlations and latent factor structure

Global genetic correlations between CRS and nine cardiovascular disease traits were estimated using LD score regression. Global genetic correlations were estimated using LD score regression (Table 2). Several significant positive correlations (p < 0.05) were identified. Notably, CRS demonstrated a significant genetic correlation with HF (rg = 0.538, p = 0.0003), All cause HF (rg = 0.526, p = 0.0003), Cardiac arrest (CA, rg = 0.533, p = 0.047), PAS (rg = 0.305, p = 0.017), PAD (rg = 0.333, p = 0.021), MI (rg = 0.365, p = 0.014) and IHD (rg = 0.247, p = 0.023), while weaker correlations were noted with AS (rg = 0.221, p = 0.134) and AP (rg = 0.145, p = 0.288).

**Table 2:** LD score regression results between CRS and various CVD traits.

Trait pair	rg	SE	p
CRS-AS	0.221	0.147	0.134
CRS-PAS	0.305	0.128	0.017^a^
CRS-PAD	0.333	0.144	0.021^a^
CRS-MI	0.365	0.149	0.014^a^
CRS-AP	0.145	0.136	0.288
CRS-IHD	0.247	0.109	0.023^a^
CRS-HF	0.538	0.147	0.0003^a^
CRS-All cause HF	0.526	0.145	0.0003^a^
CRS-CA	0.533	0.269	0.047^a^

rg, Genetic correlation; SE, Standard Error; p, p-value, ^a^ p < 0.05; CRS, Chronic Rhinosinusitis; AS, Atherosclerosis; PAS, Peripheral Atherosclerosis; PAD, Peripheral Artery Disease; HF, Heart Failure; MI, Myocardial Infarction; IHD, Ischemic Heart Disease; AP, Angina Pectoris; CA, Cardiac Arrest; CVD, Cardiovascular Disease; LD, Linkage Disequilibrium.

**Fig. 1:**
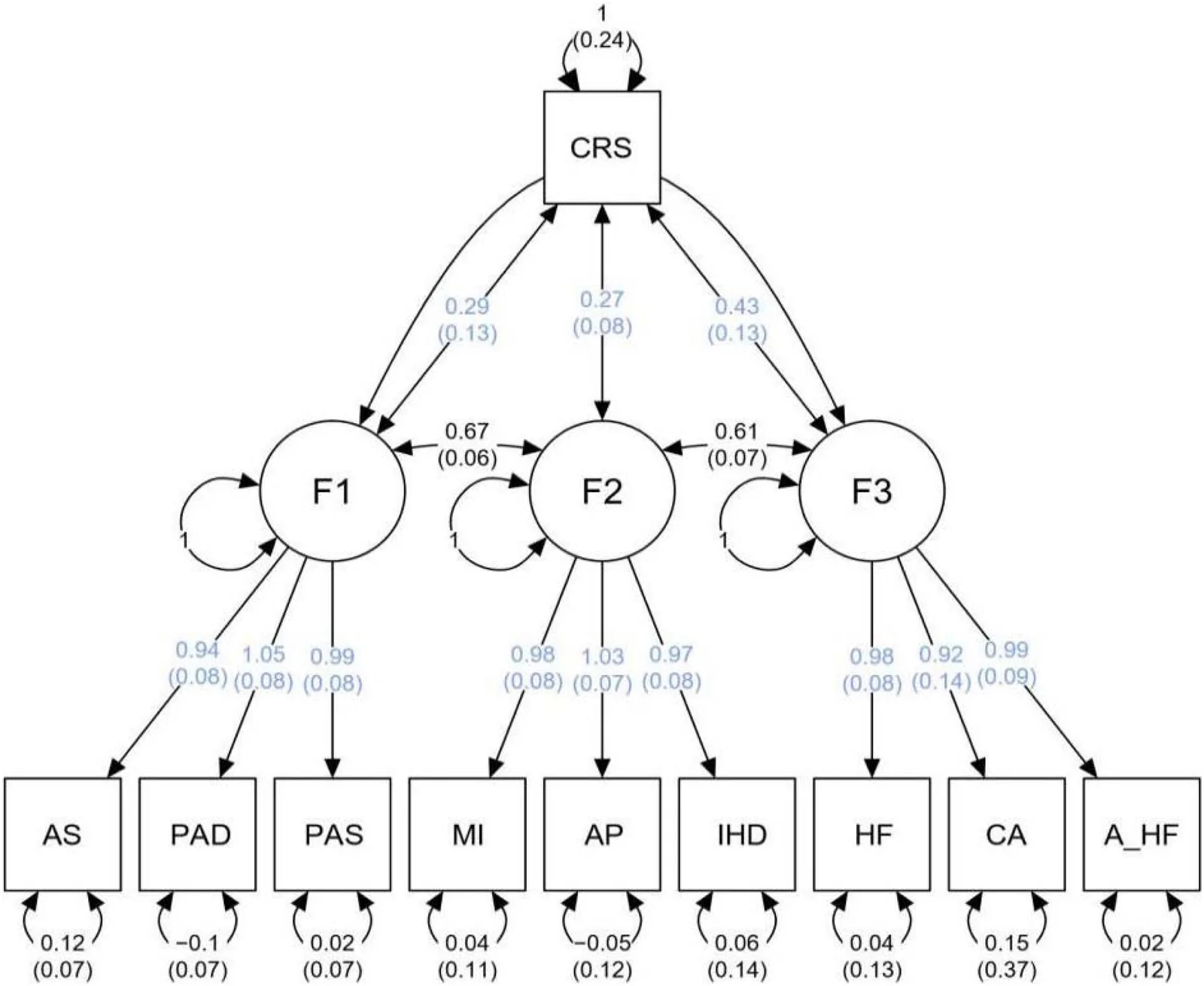
**Common factor model for CRS and CVD traits.** Path diagram with standardized factor loadings in the hierarchical model estimated using Genomic SEM. Arrows denote path regression estimates. Standardized path estimates are presented with their standard error in parentheses.

To dissect the shared genetic architecture, we fitted a hierarchical common factor model via Genomic SEM (Fig. 1). The model captured the shared genetic liability between CRS and various CVD traits as a higher-order latent factor, with excellent model fit (CFI = 0.998, SRMR = 0.059). CRS loading was fixed to 1 for identification. The shared genetic liability was modeled through three latent factors, confirming that CRS shares genetic liability with CVDs primarily through three etiological pathways in Fig. 1. F1 represented genetic risk for artery diseases, including AS, PAS and PAD (loadings: 0.94, 0.99, 1.05). F2 captured genetic risk for myocardial diseases, including MI, AP, and IHD (loadings: 0.98, 1.03, 0.97). F3 reflected genetic risk for heart failure and related conditions, comprising HF, CA and All-Cause HF (loadings: 0.98, 0.92, 0.99).

### Local genetic correlations and causal effects of CRS on peripheral artery diseases

**Fig. 2:**
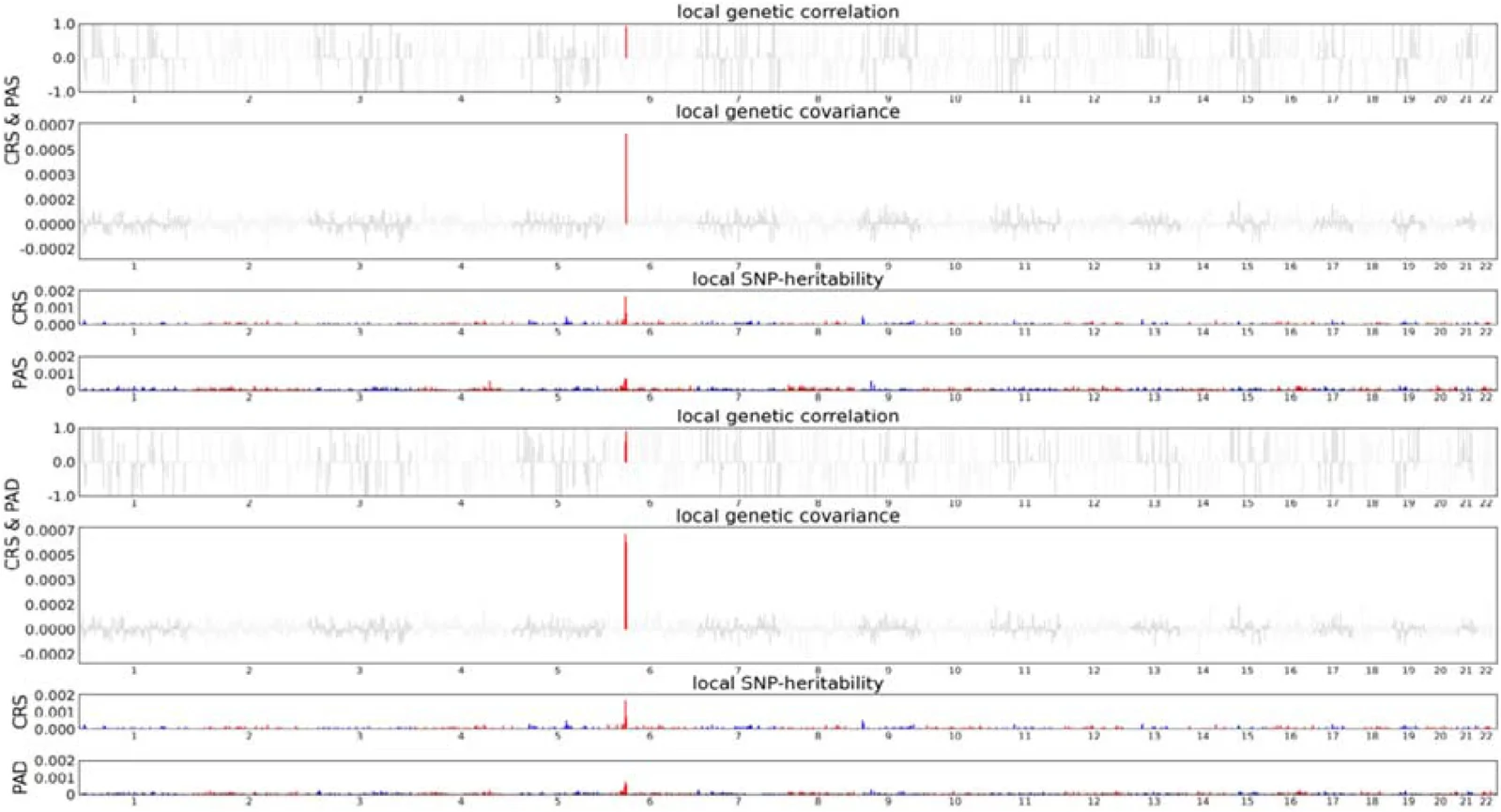
**Local genetic correlation between CRS and PAS/PAD.** Manhattan plots showed the estimates of local genetic correlation and local genetic covariance between CRS & PAS (upper three panels) and CRS & PAD (lower three panels), as well as local SNP-heritability of CRS and PAS (middle upper panels) and CRS and PAD (middle lower panels). Red bars indicate genomic regions significant after Bonferroni correctio (p = 1.50 × 10^−5^) for CRS & PAS; p = 2.52 × 10^−5^ and 1.18 × 10^−5^) for CRS & PAD.

To dissect region-specific genetic overlaps, we performed ρ-HESS analysis for CRS and nine CVD traits. Only PAD and PAS exhibited statistically significant local genetic correlations with CRS after Bonferroni corrections in Table 3 and Fig. 2. For CRS and PAS, one genomic region on chromosome 6 (chr6: 32,682,664‒33,236,497) exhibited a statistically robust association (local_rhog = 0.00062699, z = 4.3285, p = 1.50e-05). CRS and PAD showed two distinct loci on chromosome 6. The first locus (chr6: 31,571,218‒32,682,664) spanned 6,073 SNPs (local_rhog = 0.000633, z = 4.2132, p = 2.52e-05), while the second overlapped with the PAS-associated region (chr6: 32,682,664–33,236,497, 3,628 SNPs, local_rhog = 0.000574, z = 4.3813, p = 1.18e-05). Both loci showed exceptionally high statistical reliability (z > 4.2, SE < 0.00015), with the shared region (chr6: 32.68‒33.24 Mb) emerging as a genomic hotspot for CRS-PAD and CRS-PAS interactions.

**Table 3:** Local genetic correlations between CRS and peripheral artery diseases.

CHR	Str	End	Phenotype	h2CRS	h2Pheno	rg
6	32682664	33236497	PAS	0.00066926	0.00068069	0.929
6	31571218	32682664	PAD	0.0016841	0.00072865	0.572
6	32682664	33236497	PAD	0.00066926	0.00058452	0.918

h^2^, Regional Heritability; rg, Local genetic correlation coefficient; Mb, Megabase (1 × 10^6^ base pairs).

**Fig. 3:**
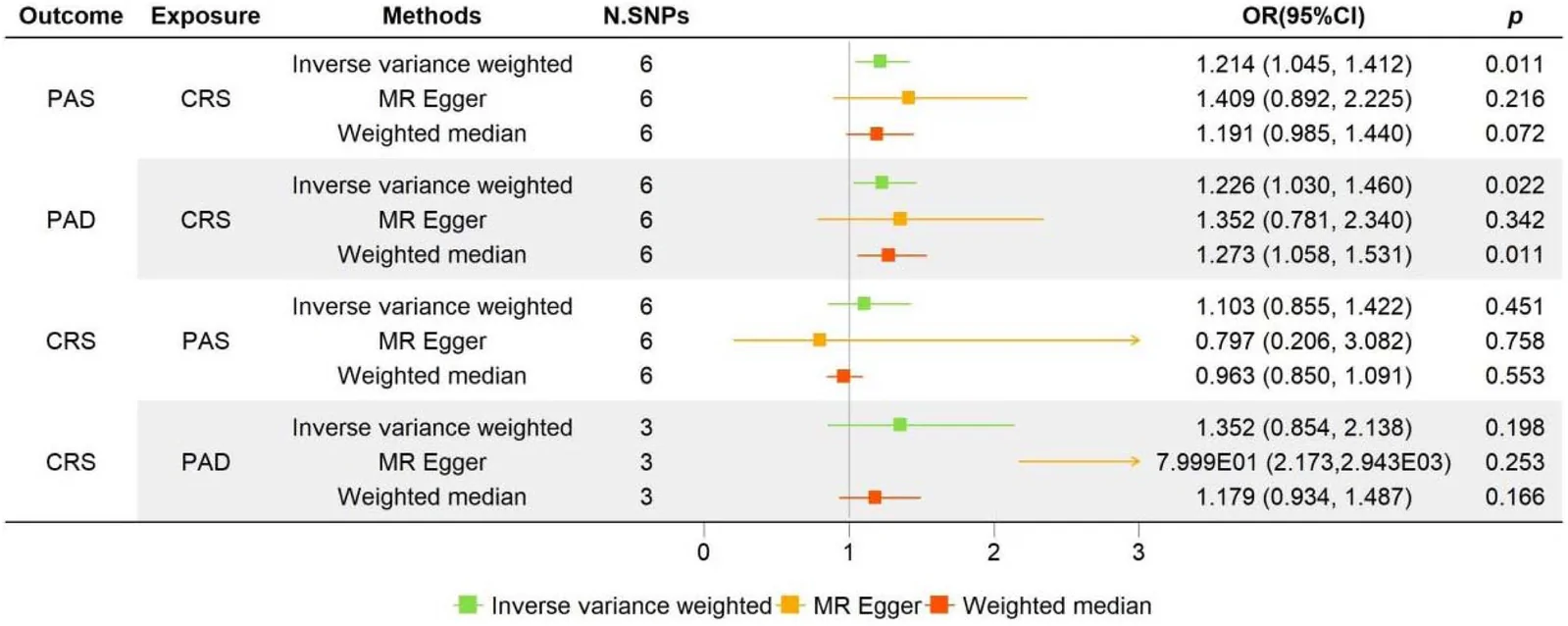
**Mendelian randomization analysis of the causal effects between CRS and PAD/PAS.** Bidirectional Mendelian randomization analysis of causal effects between CRS and PAD/PAS. Forest plot shows Odds Ratios (ORs) and 95% Confidence Intervals from Inverse-Variance Weighted (IVW), MR-Egger, and weighted median methods. N.SNPs, Number of Single Nucleotide Polymorphisms (p-value).

Building on the localized genetic correlations at chr6: 31.57‒33.24, we performed bidirectional MR to assess causal relationships between CRS and Peripheral Artery Diseases (PAS/PAD). Genetic CRS demonstrated significant causal effects on PAD and PAS through the primary Inverse-Variance Weighted (IVW) analysis. For PAD, each unit increase in genetic risk for CRS elevated disease risk by 23% (OR = 1.23, 95% CI 1.03‒1.46, p = 0.022). Similarly for PAS, CRS increased risk by 21% (OR = 1.21, 95% CI 1.04‒1.41, p = 0.011). Reverse MR analysis found no significant causal effects of PAS or PAD on CRS (IVW p > 0.05), suggesting the causal direction originates from CRS to peripheral artery diseases in Fig. 3.

Sensitivity analyses confirmed the robustness of these findings. Cochran’s Q test indicated no significant heterogeneity (p > 0.05), and leave-one-out analysis showed stable results. MR-Egger intercept tests detected no significant horizontal pleiotropy for CRS on PAS (*intercept* = −0.026, p = 0.535) or PAD (*intercept* = −0.017, p = 0.729). Cochran’s *Q* statistics further confirmed minimal heterogeneity among instruments (Table 4). 3) The MR-PRESSO global test further corroborated the absence of horizontal pleiotropy (global test p > 0.05 for all analyses) and identified no outlier SNPs. Collectively, the convergence of these stringent sensitivity analyses ‒ namely Cochran’s *Q* test, the MR-Egger intercept, and the MR-PRESSO global test ‒ coupled with consistent directional estimates across the IVW, weighted median, and MR-Egger models, provides substantial support for the robustness of our causal inferences.

**Table 4:** Results of sensitivity analysis.

Exposure	Outcome	Intercept	SE	Cochrane *Q*	DF	Cochrane p-value	p-value
CRS	PAD	−1.71E-02	4.60E-02	9.69E+00	5	8.45E-02	7.29E-01
CRS	PAS	−2.60E-02	3.83E-02	5.90E+00	5	3.16E-01	5.35E-01
PAS	CRS	5.68E-02	1.18E-01	3.48E+01	5	1.63E-06	6.56E-01
PAD	CRS	−5.83E-01	2.62E-01	2.01E+01	2	4.23E-05	2.69E-01

Parameters include MR-Egger intercept (and standard error) for pleiotropy, and Cochrane's *Q* statistic (with degrees of freedom and p-value) for heterogeneity.

### Causal gene mapping and colocalization evidence

**Fig. 4:**
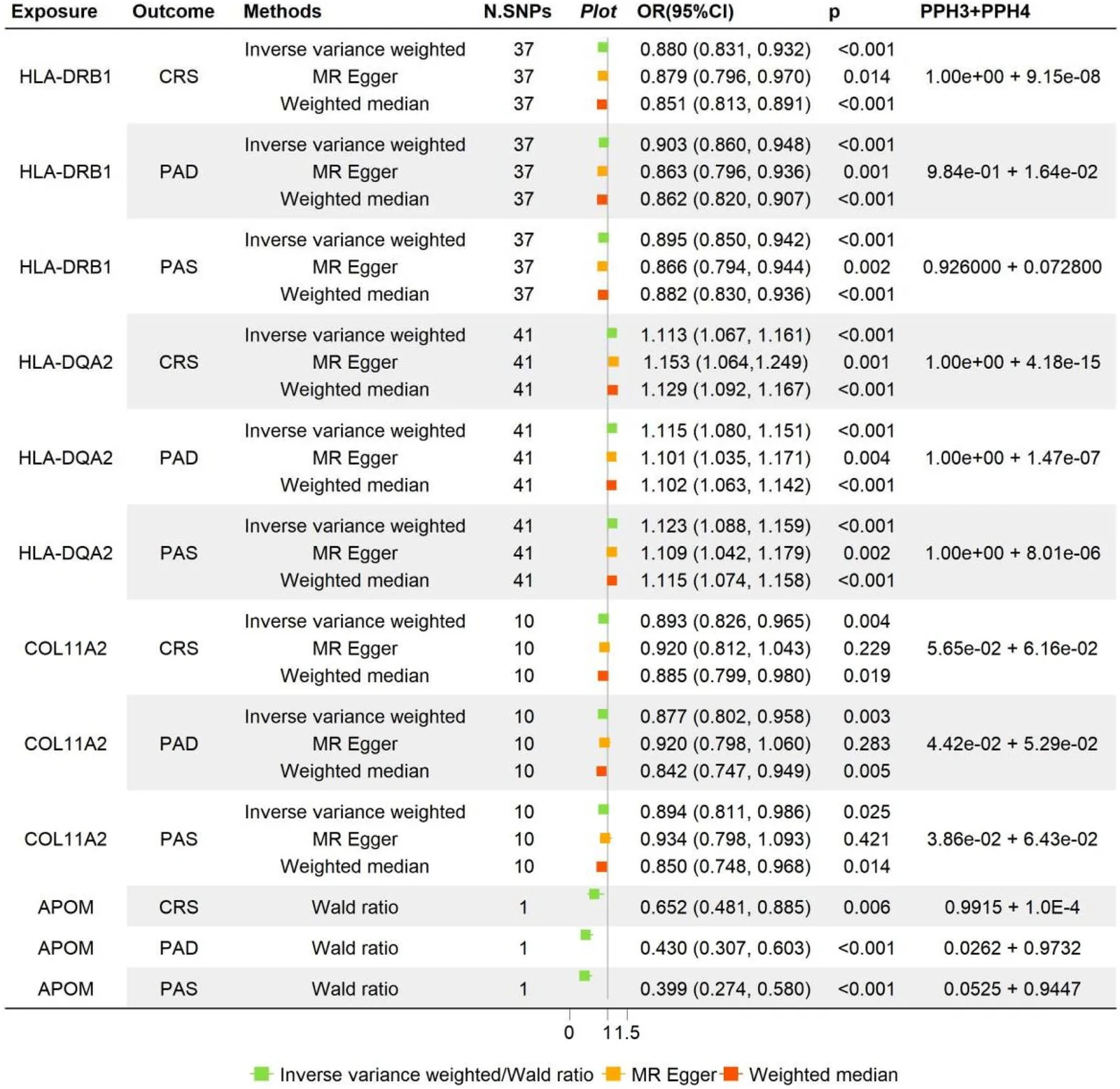
**Mendelian randomization analysis assessing causal effects of genetically predicted gene expression (eQTLs) on CRS and PAD/PAS.** The figure presents results the causal relationships between expression Quantitative Trait Loci (eQTLs) for four genes (HLA-DRB1, HLA-DQA2, COL11A2, APOM) and three diseases (CRS, PAD, PAS). Results for four genes and three diseases are shown, with Odds Ratios (ORs) from IVW, MR-Egger, and weighted median methods. The rightmost column shows posterior probabilities (PPH3 + PPH4) from Bayesian colocalization analysis. For APOM, the Wald ratio estimate is presented.

To pinpoint causal genes underlying the chr6: 31.57‒33.24 Mb hotspot, we evaluated 31 candidate genes using cis-eQTLs as instruments. The detailed are presented in Supplemental Table 3. Four genes ‒ APOM, COL11A2, HLA-DRB1, and HLA-DQA2 ‒ demonstrated significant causal effects across CRS, PAD, and PAS in Fig. 4 and Supplemental Table 4. Elevated expression of HLA-DRB1 conferred consistent protective effects across all three phenotypes, with IVW estimates demonstrating a 12% reduction in CRS risk (OR = 0.88, 95% CI 0.83‒0.93, p = 1.38 × 10^−5^), 10% lower PAD risk (OR = 0.90, 95% CI 0.86‒0.95, p = 3.81 × 10^−5^), and 11% decreased PAS risk (OR = 0.89, 95% CI 0.85‒0.94, p = 2.01 × 10^−5^). Similarly, increased APOM expression showed significant protection against all three conditions, reducing CRS risk by 35% (Wald ratio OR = 0.65, 95% CI 0.48‒0.89, p = 0.006), PAD risk by 57% (Wald ratio OR = 0.43, 95% CI 0.31‒0.60, p = 1.00 × 10^−6^), and PAS risk by 60% (Wald ratio OR = 0.40, 95% CI 0.27‒0.58, p = 1.47 × 10^−6^). COL11A2 expression also showed protective effects, most prominently a 12% reduction in PAD risk (IVW OR = 0.88, 95% CI 0.80‒0.96, p = 0.003). In stark contrast, elevated HLA-DQA2 expression increased disease susceptibility, elevating CRS risk by 11% (IVW OR = 1.11, 95% CI 1.07‒1.16, p = 5.18 × 10^−7^) with consistent risk effects observed for peripheral artery diseases. To assess potential horizontal pleiotropy bias, Egger regression intercept tests were performed. No statistically significant evidence of directional horizontal pleiotropy was found for any of the associations between HLA-DRB1, HLA-DQA2, or COL11A2 and the outcomes PAD, PAS, or CRS (all Egger intercept p-values > 0.05). Full results are provided are presented in Supplemental Table 5.

Colocalization analyses revealed significant genetic associations for HLA-DRB1 and HLA-DQA2 across three traits (CRS, PAD, PAS) under the combined posterior probability threshold (PPH3 + PPH4 > 0.8), consistent with region-specific but distinct causal variants. Notably, APOM exhibited dichotomous mechanisms: CRS showed H3-driven association (PPH3 = 0.9915, PPH4 = 1.0e^−4^), while PAD and PAS demonstrated strong H4 evidence (PAD: PPH3 = 0.0262, PPH4 = 0.9732, candidate SNP: rs3115663; PAS: PPH3 = 0.0525, PPH4 = 0.9447, candidate SNP: rs3115663), suggesting shared causal variants for vascular traits but distinct variants for CRS. In contrast, COL11A2 associations were uniformly driven by Hypothesis 1 (H1) across traits (CRS: PPH1 = 0.882; PAD: PPH1 = 0.903; PAS: PPH1 = 0.897), with minimal H3/H4 contributions (combined PPH3 + PPH4 ≤ 0.118), indicating variant effects restricted to COL11A2 without cross-phenotype colocalization. Collectively, these results confirm robust region-specific genetic causality with divergent molecular mechanisms underlying each gene-trait interaction in Fig. 4.

Functional pathway analysis of the four candidate genes (APOM, COL11A2, HLA-DRB1, HLA-DQA2) indicated their extensive involvement in key biological modules, including immunity, metabolism, inflammation, and ciliary motility. HLA-DRB1 and HLA-DQA2 demonstrated predominant associations with immune responses, highlighting their roles in antigen presentation, while HLA-DRB1 also showed connections to metabolic and ciliary motility pathways. APOM was implicated in inflammatory, metabolic, and immune processes, particularly through interactions related to lipid transport. COL11A2 was primarily linked to structural genes and inflammatory pathways (Supplemental Table 6).

### In vitro validation of endothelial cell response to inflammatory stimuli

**Fig. 5:**
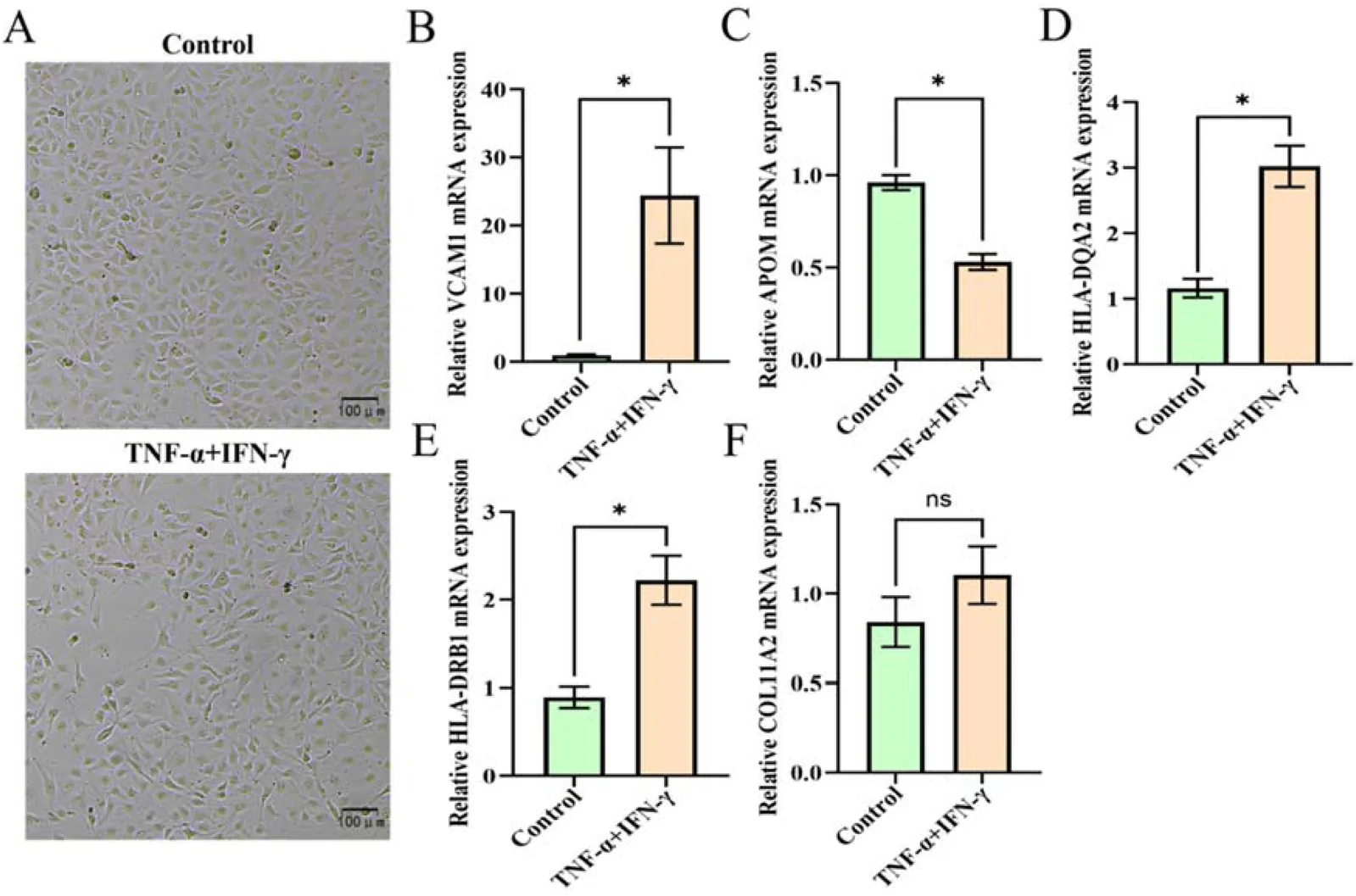
**Endothelial cell responses to inflammatory stimuli in vitro.** (A) HUVECs cultured in standard medium (Control) or supplemented with TNF-α and IFN-γ. Scale bar = 100 μm. (B‒F) RT-qPCR analysis of relative mRNA expression for (B) VCAM-1, (C) APOM, (D) HLA-DQA2, (E) HLA-DRB1, and (F) COL11A2. Data are presented as mean ± SD of three independent experiments (* p < 0.05).

To functionally validate the potential role of genetic loci identified in our analysis, we conducted an in vitro experiment using HUVECs. HUVECs serve as a well-established model system for studying vascular endothelial inflammation, immune activation, and dysfunction, which are central to the pathogenesis of cardiovascular diseases. Following a 48-h exposure to TNF-α and IFN-γ, utilized to model the systemic endothelial inflammation hypothesized in our study, HUVECs exhibited morphological alterations. Compared to the typical cobblestone-like confluent monolayer in the control group, cytokine-treated cells displayed an elongated and irregular phenotype indicative of endothelial activation (Fig. 5A). To confirm the induction of a pro-atherosclerotic state, we quantified the expression of VCAM-1, a hallmark of endothelial dysfunction. Inflammatory stimulation elicited a significant upregulation of VCAM-1 mRNA levels (p < 0.05), validating the experimental model (Fig. 5B). Subsequent evaluation of the four candidate genes revealed that APOM, identified as protective in our genetic analysis, was significantly downregulated under inflammatory stress (p < 0.05, Fig. 5C). In contrast, the expression of the risk-associated gene HLA-DQA2 was significantly elevated following cytokine treatment (p < 0.05, Fig. 5D). HLA-DRB1, was also significantly upregulated in the inflammatory milieu (p < 0.05, Fig. 5E). Finally, while COL11A2 expression exhibited a slight upward trend post-stimulation, this change did not reach statistical significance (Fig. 5F).

## Discussion

### Principal findings and genetic architecture

Our LD score regression analyses revealed significant positive global genetic correlations between CRS and several CVD traits, most notably PAS, PAD, HF, CA, MI, and IHD, indicating common biological pathways. The hierarchical common factor model via Genomic SEM further elucidated this shared architecture into three latent factors: F1 (artery diseases: AS, PAS, PAD), F2 (myocardial diseases: MI, AP, IHD), and F3 (heart failure and related conditions: HF, CA, All-Cause HF). This structure indicates that the genetic predisposition linking CRS to CVDs operates through three distinct etiological pathways, potentially reflecting mechanisms such as vascular inflammation, myocardial stress responses, and systemic immune dysregulation.

### Local genetic correlations and causal inference

Focusing on specific genomic regions, ρ-HESS analysis pinpointed significant local genetic correlations specifically between CRS and Peripheral Artery Diseases (PAD, PAS) within a defined locus on chromosome 6 (chr6: 31.57–33.24 Mb). This region encompasses the Major Histocompatibility Complex (MHC), implicating immune-mediated mechanisms as a critical link between chronic sinonasal inflammation and peripheral arterial pathologies, consistent with reports that MHC variants influence endothelial dysfunction and epithelial barrier integrity.^12^^,^^34^^,^^35^, ^36^, ^37^ Subsequent bidirectional MR analyses provided robust evidence for a unidirectional causal effect: genetic predisposition to CRS significantly increases the risk of developing both PAD and PAS. This finding has significant clinical implications, suggesting that effective management of CRS could potentially mitigate the risk of subsequent peripheral vascular complications.

Within the identified chr6 hotspot, cis-MR implicated four genes-APOM, COL11A2, HLA-DRB1, and HLA-DQA2-as causally influencing CRS, PAD, and PAS. Dysregulation of antigen presentation or T-cell activation mediated by these molecules provides a plausible mechanism linking chronic mucosal inflammation in CRS with systemic vascular inflammation and atherosclerosis. The protective role of HLA-DRB1 may reflect specific alleles that promote immune tolerance.^38^^,^^39^ The opposing effects of HLA-DRB1 and HLA-DQA2, key components of the MHC class II complex, highlight the critical balance within immune pathways.^40^ APOM, a component of High-Density Lipoprotein (HDL), links lipid transport and immunomodulation. Its potent protective effect strongly supports the role of lipid metabolism and anti-inflammatory HDL functions in mitigating peripheral artery disease risk, with implications for CRS via inflammatory modulation.^41^ COL11A2, encoding a fibrillar collagen, is essential for extracellular matrix integrity. Its protective effect suggests that maintaining vascular structural integrity may represent a shared pathway against CRS-related vascular complications, warranting further investigation.^42^

### Potential biological and pathophysiological mechanisms

Beyond genetic susceptibility, the mechanistic interplay between CRS and PAD can be contextualized through established experimental and clinical pathology studies. Chronic sinonasal inflammation in CRS potentially induces a systemic spillover of pro-inflammatory cytokines, driving remote endothelial dysfunction ‒ an early atherosclerotic event.

Crucially, experimental models demonstrate that APOM, the physiological carrier for Sphingosine-1-Phosphate (S1P), maintains vascular endothelial barrier integrity and downregulates pro-inflammatory adhesion molecules (VCAM-1)^43^ While the broader apolipoprotein family (e.g., APOA2, A2M, APOE) actively participates in sinonasal inflammation and tissue remodeling.^44^, ^45^, ^46^ Our in vitro model aligns with this upstream genetic prediction: under inflammatory stress mimicking the CRS systemic milieu, HUVECs exhibited profound APOM downregulation and concurrent VCAM-1 upregulation. This suggests genetic deficiency in APOM expression inherently predisposes individuals to PAD, a vascular vulnerability that is severely exacerbated when CRS-derived systemic inflammation actively depletes the remaining protective APOM pool in the endothelium.

Similarly, recent clinical histological and single-cell analyses reveal that under chronic inflammatory stress, vascular endothelial cells aberrantly upregulate MHC-II expression, directly triggering localized T-cell activation and accelerating atherogenesis.^47^^,^^48^ Concurrently, persistent immune stimulation in CRS causes a systemic imbalance of Th1, Th2, and Th17 subsets.^49^ The systemic release of cytokines from these dysregulated T-cells is a recognized driver of vascular inflammation and atherogenesis. Our experimental observations align with the established pathological interactions between systemic inflammation and vascular antigen presentation. The inflammatory stimuli significantly upregulated the risk gene HLA-DQA2, which is consistent with its active pathogenic role in exacerbating antigen presentation at the vascular wall. Furthermore, the protective allele HLA-DRB1 was also significantly upregulated. Rather than acting as a pathogenic driver, contemporary immunological evidence suggests this represents a compensatory, negative-feedback regulatory mechanism.^50^ However, we postulate that this transient endothelial defense mechanism is continuously exhausted and ultimately overwhelmed by the chronic progression of CRS, leading to unabated vascular inflammation. Finally, while COL11A2 demonstrated protective effects in MR, it lacked cross-phenotype colocalization and significant in vitro responsiveness. This implies that COL11A2 likely protects against vascular complications primarily by maintaining basal extracellular matrix structural integrity, rather than acting as a dynamic responder to acute systemic inflammation.^51^ Collectively, these findings validate that CRS and PAD are causally linked through specific upstream perturbations in immune-metabolic (HLA/APOM) gene regulation.

### Limitations and future directions

Several limitations should be considered. Firstly, the study population was restricted to individuals of European descent. Given that allele frequencies and LD structures vary globally, necessitating multi-ancestry GWAS and MR studies. Secondly, aggregating CRS cases without stratifying by clinical endotypes may mask subtype-specific cardiovascular genetic predispositions, requiring future deeply phenotyped cohorts. Most importantly, the current in vitro validation utilized a HUVEC monoculture. While our HUVEC model provides a critical proof-of-concept for acute endothelial responses, it cannot fully capture systemic sinonasal-vascular crosstalk. Given our primary focus on establishing a causal genetic architecture, exhaustive mechanistic dissection via CRISPR-edited co-cultures or in vivo models falls beyond the current scope, warranting dedicated future investigations. Furthermore, While MR provides causal evidence, it cannot replace longitudinal clinical observations. Prospective clinical trials are needed to track CRS patients for subclinical markers of arterial stiffness and to determine if effective sinonasal management can reduce long-term PAD risk.

## Conclusion

In conclusion, this integrative genetic study demonstrates a significant shared genetic liability and a causal relationship between CRS and peripheral artery diseases. We identified a critical genomic hotspot on chromosome 6 and pinpointed specific causal genes (HLA-DRB1, HLA-DQA2, APOM, COL11A2) whose expression levels modulate disease risk. The in vitro model functionally corroborated these genetic predictions, demonstrating that systemic inflammatory stress profoundly disrupts lipid-mediated vasoprotection (APOM) and exacerbates aberrant antigen presentation (HLA-DQA2) at the endothelial level. Collectively, our findings underscore the systemic implications of CRS and highlight potential therapeutic targets for mitigating cardiovascular risk in affected individuals.

## ORCID ID

Jiaoyu He: 0000-0003-4585-1354

Weigang Gan: 0000-0003-0582-946X

Binbin Wang: 0009-0001-9049-3026

Feng Liu: 0000-0003-2866-2340

## Declaration of competing interest

The authors declare no conflicts of interest.
