# Supplementary material for: Unraveling causal links between chronic rhinosinusitis and peripheral artery diseases: insights from genetic correlations through genome-wide association studies
Source: Braz J Otorhinolaryngol. 2026 Sep 2;92(5):101891. doi: 10.1016/j.bjorl.2026.101891 (PMC13563561; doi:10.1016/j.bjorl.2026.101891)
Supplement: Supplementary file 1 [file mmc1.docx]

BJORL-D-25-00397_Supplementary Material

**Supplemental Table 1** SNP information.

**Supplemental Table 2** GWAS information.

**Supplemental Table 3** eQTL information.

**Supplemental Table 4** Mendelian randomization results.

**Supplemental Table 5** eQTL MR horizontal pleiotropy test results.

**Supplemental Table 6** Functional pathway analysis of candidate genes.
